# The Roles of HIF-1α in Radiosensitivity and Radiation-Induced Bystander Effects Under Hypoxia

**DOI:** 10.3389/fcell.2021.637454

**Published:** 2021-03-25

**Authors:** Jianghong Zhang, Yuhong Zhang, Fang Mo, Gaurang Patel, Karl Butterworth, Chunlin Shao, Kevin M. Prise

**Affiliations:** ^1^Institute of Radiation Medicine, Fudan University, Shanghai, China; ^2^Patrick G Johnston Centre for Cancer Research, Queen’s University Belfast, Belfast, United Kingdom

**Keywords:** radiation, hypoxia, bystander, HIF1 alpha, micronuclei, hepatoma cells

## Abstract

Radiation-induced bystander effects (RIBE) may have potential implications for radiotherapy, yet the radiobiological impact and underlying mechanisms in hypoxic tumor cells remain to be determined. Using two human tumor cell lines, hepatoma HepG2 cells and glioblastoma T98G cells, the present study found that under both normoxic and hypoxic conditions, increased micronucleus formation and decreased cell survival were observed in non-irradiated bystander cells which had been co-cultured with X-irradiated cells or treated with conditioned-medium harvested from X-irradiated cells. Although the radiosensitivity of hypoxic tumor cells was lower than that of aerobic cells, the yield of micronucleus induced in bystander cells under hypoxia was similar to that measured under normoxia indicating that RIBE is a more significant factor in overall radiation damage of hypoxic cells. When hypoxic cells were treated with dimethyl sulfoxide (DMSO), a scavenger of reactive oxygen species (ROS), or aminoguanidine (AG), an inhibitor of nitric oxide synthase (NOS), before and during irradiation, the bystander response was partly diminished. Furthermore, when only hypoxic bystander cells were pretreated with siRNA hypoxia-inducible factor-1α (HIF-1α), RIBE were decreased slightly but if irradiated cells were treated with siRNA HIF-1α, hypoxic RIBE decreased significantly. In addition, the expression of HIF-1α could be increased in association with other downstream effector molecules such as glucose transporter 1 (GLUT-1), vascular endothelial growth factor (VEGF), and carbonic anhydrase (CA9) in irradiated hypoxic cells. However, the expression of HIF-1α expression in bystander cells was decreased by a conditioned medium from isogenic irradiated cells. The current results showed that under hypoxic conditions, irradiated HepG2 and T98G cells showed reduced radiosensitivity by increasing the expression of HIF-1α and induced a syngeneic bystander effect by decreasing the expression of HIF-1α and regulating its downstream target genes in both the irradiated or bystander cells.

## Introduction

Hypoxic regions are a common feature of most solid tumors, have radioresistant biological effects, and represent the most aggressive fraction of a tumor (Horsman et al., 2012; Peitzsch et al., 2017; Busk et al., 2020). Recent experimental evidence has shown that cells may respond directly to radiation exposure or through communicated transmissible factors capable of inducing cellular responses in non-irradiated cells, which is referred to as the radiation-induced bystander effect (RIBE) (Shao et al., 2003; Kamochi et al., 2008; Lorimore et al., 2008; Shao et al., 2008a; Zhu et al., 2008; Heeran et al., 2019). The RIBE has been demonstrated in a range of experimental systems and been shown to induce DNA point mutations (Gaziev et al., 2008; Nikitaki et al., 2016), DNA double-strand breaks (Shikazono et al., 2009), genomic instability (Behar, 2008; Lorimore et al., 2008), micronucleus formation (Shao et al., 2005, 2006; Xie et al., 2015), change in gene expression (Xie et al., 2016), apoptosis, and cell killing (Yasui et al., 2017). There may be a close interrelationship between cellular bystander responses and systemic tissue responses to radiation exposure including abscopal effects and the involvement of immune signaling (Formenti and Demaria, 2009; Prise and O’Sullivan, 2009; Griffin et al., 2020a,b). Despite this, the role of RIBE in radiotherapy and cancer risks associated with environmental, occupational, and medical exposures remains to be fully elucidated. Furthermore, the impact of factors within the tumor microenvironment including hypoxia has not yet been determined.

The precise mechanisms underlying RIBE, especially under hypoxic conditions, are still unclear. There is evidence that a range of bystander signaling molecules, including free radicals (Dong et al., 2015), protein factors (Shao et al., 2008a; Xie et al., 2016), calcium ions (Shao et al., 2006), hormones, microRNA (Palayoor et al., 2014), and extracellular vesicles (EVs) (Bewicke-Copley et al., 2017), can be produced from irradiated cells and play a role in the induction of bystander responses. In addition, gap-junctional intercellular communication (GJIC) has been shown to play an important role in the bystander response of normal cells which have fully functional GJIC (Shao et al., 2003; Frank et al., 2006). RIBE can be triggered by irradiated tumor cells so that heritable damages are induced in surrounding tumor and normal cells. For example, we found that when a small fraction of glioblastoma cells T98G were targeted with localized ions from a microbeam, additional micronucleus could be induced in the neighboring cells of either T98G or normal human fibroblasts (Mayer et al., 2012), which suggests that bystander response may have two potential impacts on radiotherapy by increasing the efficacy of tumor cell killing but simultaneously increasing the secondary cancer risk via the production of genetic damage occurs in normal bystander cells.

It has been noted that hypoxia can increase radioresistance of malignant solid tumors through conspecific biological mechanisms (Moeller et al., 2007; Li et al., 2010; Sun et al., 2015). A key mechanism is via the transcription factor, hypoxia-inducible factor 1 (HIF-1). Extensive research has shown that increasing intratumoral hypoxia and HIF-1 activity correlate with incidences of both tumor recurrence and distant tumor metastasis as well as a poor prognosis and after radiation therapy. HIF-1 is able to promote the growth of endothelial cells after radiotherapy by inducing the expression of key downstream genes, including vascular endothelial cell growth factor (VEGF) (Ahn et al., 2014), carbonic anhydrase (CA9) (Chiche et al., 2009; Logsdon et al., 2016), and glucose transporter (GLUT1) (Dungwa et al., 2011), thus promoting the overall tumor radioresistance. HIF-1 is a heterodimeric transcription factor consisting of both an α-subunit (HIF-1α) and a β-subunit (HIF-1β). Its activity is mainly dependent on the stability of HIF-1α, which becomes relatively stable under hypoxia, then interacts with HIF-1β to form the HIF-1 active heterodimer. This binds to its enhancer sequence, the hypoxia-responsive element (HRE), and induces the expression of key genes related to adapting cellular metabolism to hypoxia, escaping or improvement in hypoxia, and the resistance of malignant tumor to chemo and radiation therapy (Prise and O’Sullivan, 2009; Semenza, 2009; Noman et al., 2019).

Here, we demonstrate a significant bystander response under hypoxic conditions in two cell models, which is comparable to that observed under oxic conditions, despite the reduction in direct effect. This highlights an overall increased role for bystander signaling under hypoxic conditions. A significant role for ROS and NO in mediating the response is reported. We also report that the increased expression of HIF-1α and its downstream proteins in hypoxic-irradiated cells leads to an increase of radioresistance but induces a bystander effect via downregulating HIF-1α expression in hypoxic non-irradiated bystander cells. These could have future benefits for the development of therapeutic strategies for treatment of hypoxic tumors.

## Materials and Methods

### Cell Culture and Drugs

Human hepatoma cells HepG2 and human glioblastoma cells T98G were obtained from Cancer Research UK, London, and maintained in Dulbecco’s minimum essential medium (DMEM, Gibco) supplied with 10% fetal calf serum (FCS, Gibco), 0.01% sodium pyruvate (Sigma), 2 mM L-glutamine, 100 units/ml penicillin, and 100 μg/ml streptomycin in humidified 95% air and 5% CO_2_ at 37 °C. For hypoxic incubation, cells were maintained in an InvivO2-400 hypoxia workstation (Ruskinn Technology Ltd., United Kingdom) with 94.9% N_2_, 5% CO_2_, and 0.1% O_2_ for at least 12 h. Target cells were placed into a sealed aluminum box at 37 °C with 95 % N_2_ and 5% CO_2_ for 4 h; after irradiation, target cells were transferred into the hypoxic workstation for further treatment. Bystander cells were cultured or treated while still in the hypoxia workstation.

Dimethyl sulfoxide (DMSO), aminoguanidine (AG), cobalt chloride (CoCl_2_), cytochalasin B (CB), and 7-aminoflavone (AF) were purchased from Sigma (United Kingdom). HIF-1α siRNA (h2, sc-44225) and control siRNA were purchased from Santa Cruz Biotechnology, Inc (United Kingdom). Lipofectamine 2000 (Cat. No. 11668-019) was from Invitrogen (United Kingdom). Opti-MEM-I was from Gibco (United Kingdom).

### Cell Irradiation

Log-phase cells were seeded and incubated overnight before being incubated in the hypoxic workstation for 12 h. One hour before irradiation, the culture medium was replaced by a serum-free and hypoxic medium containing 10 mM HEPES and the culture dishes were put into a custom-built aluminum box flushed with 95% N_2_ and 5% CO_2_ (BOC, United Kingdom) for cell irradiation. At room temperature, cells were irradiated with 225 kVp X-rays (2-mm copper-filtered, X-Rad 225, Precision X-ray Inc., United States) at a dose rate of 0.59 Gy/min.

In the experiments where cells were treated before irradiation with 1% DMSO or 20 μM AG for 1 h, after irradiation, the medium was immediately replaced by a complete medium for further experiments.

### Generation of Conditioned Medium for Cell Treatment

After irradiation, the medium was changed immediately. Two hours later, the conditioned medium was collected from irradiated cells and filtered through a 0.2-μm filter. Then, non-irradiated cells were treated with the conditioned medium for 24 h with the following two methods. Group one (N_2_→N_2_), bystander hypoxic cells were treated with the conditioned medium generated from cells irradiated under hypoxia. Group two (O_2_→O_2_), normoxic bystander cells were treated with the conditioned medium harvested from irradiated normoxic cells.

### Cell Co-culture

Under either normoxic or hypoxic condition, cells growing on one coverslip were irradiated with X-rays and were then co-cultured for 24 h with the same number of non-irradiated cells growing on another coverslip within the same culture dish. After this co-culture, micronucleus and cell survival were assayed in the non-irradiated cells.

### Treatment of Reagents

For chemical hypoxic treatment, normoxic HepG2 or T98G cells were treated with 100 μM CoCl_2_ for 4 h before irradiation. In specific experiments, hypoxic-irradiated cells were treated with 1% DMSO or 20 μM AG, an inhibitor for NO, 1 h before irradiation. AF was used to inhibit HIF-1α mRNA expression and HIF-1α protein accumulation, and hypoxic-irradiated or bystander cells were treated with 5 μM AF for 4 h before irradiation or conditioned-medium treatment, respectively. Transient inhibition of HIF-1α was carried out by transfection with HIF-1α siRNA or control siRNA-A using Lipofectamine^TM^ 2000 according to the manufacturer’s instruction. After irradiation, the medium was immediately replaced by a complete medium on irradiated cells for further experiments.

### Micronucleus Assay

Micronucleus induction was used as an endpoint for bystander damage. The frequency of micronucleus formation was measured using the cytokinesis-block technique (Fenech and Morley, 1986). After conditioned-medium treatment or co-culture procedure, the cells were treated with 1 μg/ml CB for 28 h, washed with PBS, fixed with methanol and acetic acid (9:1, v/v) for 20 min, stained with 10 μg/ml Hoechst 33342 plus 10 μg/ml acridine orange for 5 min, and washed in water. After air drying, the cells were mounted with Mount Medium (Sigma, United Kingdom) then at least 1000 binucleated cells were observed for micronuclei with a fluorescence microscope. The micronucleus yield (Y_*MN*_) was calculated as the ratio of the number of micronuclei to the number of binucleated cells.

### Cell Survival Assay

The irradiated HepG2 cells and T98G cells were trypsinized and replated on 60-mm dishes at appropriate dilutions immediately after irradiation with 0, 2, 4, or 8 Gy X-rays and incubated for 10–14 days at 37°C in a humidified atmosphere containing 5% CO_2_ and 95% air. To measure viability in bystander cells, cells were treated with a conditioned medium for 24 h, then treated as above. The colonies from irradiated or bystander populations were fixed with 10% formalin, stained with 1% methylene blue, and the colonies containing ≥50 cells were counted to quantify clonogenic cell survival. Survival fraction was calculated as the ratio of the plating efficiency of the irradiated cells to the plating efficiency of cells which had not been irradiated (controls). Data from three separate experiments are presented as the mean ± SEM, and the error bars for all survival data represent the 95% confidence intervals for normalized data points as calculated by Fieller’s theorem (Gupta et al., 1996). The cell survival curves were fitted with the multitarget single-hit mode of survival fraction = 1 – (1 – exp(-D/D_0_))^N by optimizing variable parameters D_0_ and N. A modified OER (oxygen enhancement ration) was calculated as the ratio of D_0_ at hypoxia as at ambient oxygen tension, i.e., OER = D_0_ (hypoxia)/D_0_ (normoxia).

### Western Blotting

Cultured cells were harvested after the reported treatments (as described as above), washed with cold PBS on ice, and lysed with RIPA lysis buffer for protein extraction. After being denatured at 100°C for 10 min, aliquots of protein samples (20 μg) were separated by electrophoresis on an SDS-polyacrylamide gel (4% pycnotic gel and 10% separation gel, Bio-Rad Laboratories, Inc), then transferred onto a polyvinylidene difluoride membrane (Millipore Corporation), blocked for 1 h with 5% skimmed milk in 0.05% Tris-buffered saline/Tween (TBST), and incubated with a specific primary antibody (diluted 1:1000 in blocking buffer for the rabbit anti-HIF-1α; Cell Signaling Technology, London, United Kingdom), 1:500 for the rabbit anti-CA9 (Abcam, Cambridge, United Kingdom), 1:1000 for the mouse anti-VEGF, 1:1000 for mouse anti-Glut 1, and 1:1000 for mouse anti-β-actin (Abcam) overnight at 4°C. Then, the membrane was washed 3× with TBST at room temperature for 10 min and incubated with a secondary antibody (HRP-conjugated anti-mouse IgG or anti-rabbit IgG; 1:5000, Abcam) for 1 h. The membrane was detected by the enhanced chemiluminescence system (ECL Advance, Amersham Biosciences) after several washes, and the protein image was recorded using a BIO-RAD ChemiDoc XRS and analyzed using Quantity One software (Bio-Rad, Hercules, CA, United States).

### Statistical Analysis

Statistical analysis was performed on the means of data obtained from at least three independent experiments. Three replicates were counted for each data point to quantify the micronucleus induction. All results are presented as mean ± SEM, analyzed with an ANOVA test. Statistical significance was defined as *P <* 0.05.

## Results

### Hypoxia-Induced Radioresistance

**FIGURE 1:**
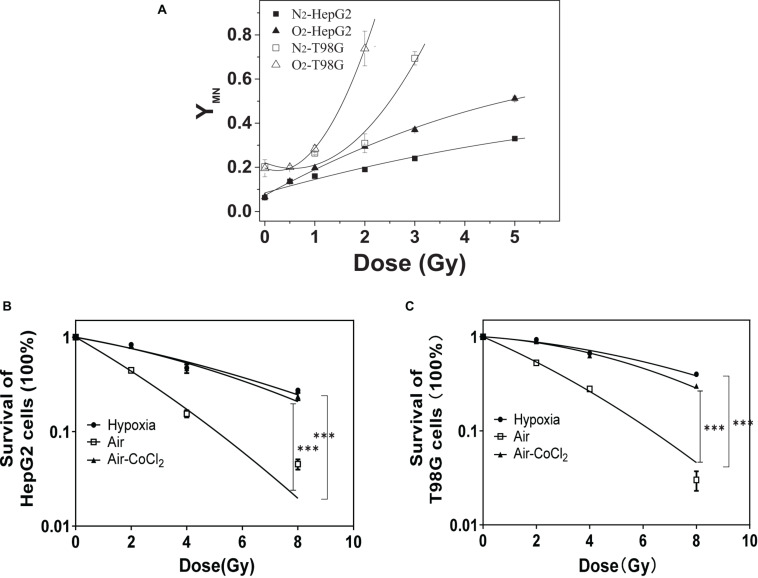
The role of hypoxia on radioresistance in HepG2 and T98G cells. The micronucleus yields of irradiated HepG2 cells and T98G cells (with 0, 0.5, 1, 2, 3, or 5 Gy X-ray) were detected under normoxic or hypoxic conditions **(A)**. Cell survival was analyzed for HepG2 cells **(B)** and T98G cells **(C)** under normoxic, hypoxic, or 100 μM cobalt chloride (CoCl_2_) pretreatment. ***p < 0.01 between indicated groups.

Chromosomal damage following direct irradiation was determined by measuring micronucleus induction after cell nuclear division. Figure 1A illustrates the yields of micronucleus in both HepG2 and T98G cell lines as a function of dose under normoxic and hypoxic conditions. T98G cells had higher yields of micronucleus under both conditions compared to HepG2 cells. For both cell lines, normoxic cell radiosensitivity was significantly higher than hypoxic cells. When the dose was 2 Gy, the yield of micronucleus in normoxic cells to the yield of micronucleus in hypoxic cells ratio was 1.6 and 2.4 for HepG2 cells and T98G cells, respectively. The radiosensitivity of hypoxic cells was increased under hypoxic conditions (Figures 1B,C). The OER of HepG2 and T98G cell was 2.56 and 2.66, respectively. Treatment of cells with CoCl_2_ gave the OERs of 2.41 and 2.26 in HepG2 and T98G cells, respectively. These results clearly demonstrate that hypoxia induces significant radioresistance.

### RIBE Played an Important Role in Radiation Damage Under Hypoxia Conditions

**FIGURE 2:**
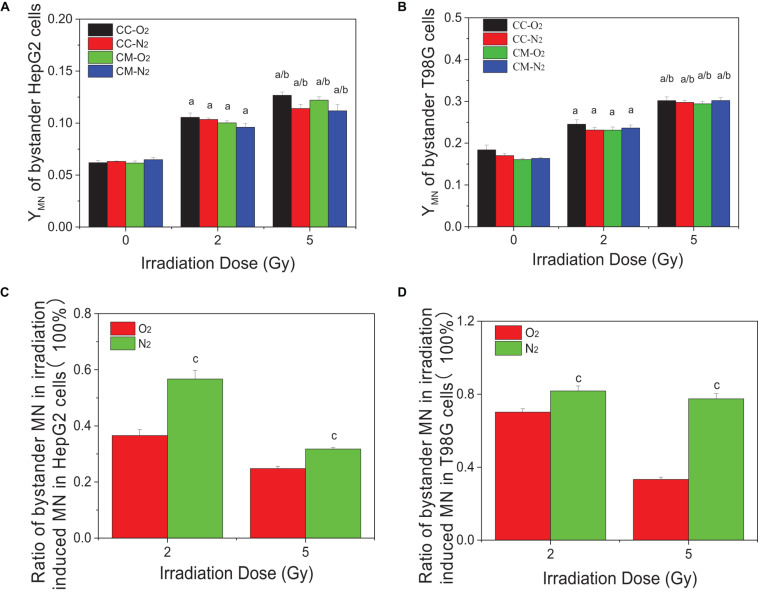
Micronucleus induction in non-irradiated bystander cells of HepG2 and T98G under hypoxic and normoxic conditions. The bystander cells were either co-cultured with irradiated conspecific cells (CC-O_2_ and CC-N_2_) or treated with the conditioned medium (CM-O_2_ and CM-N_2_) harvested from irradiated conspecific cells under the same oxygen conditions. The ratio of bystander micronucleus **(A,B)** to radiation-induced micronucleus of HepG2 cells **(C)** and T98G cells **(D)** under hypoxic and normoxic conditions was plotted. a, *p* < 0.05 compared with the 0-Gy group; b, *p* < 0.05 compared with the 2-Gy group; c, *p* < 0.05 compared with the normoxic group.

Figures 2A,B show that the micronucleus yields for both non-irradiated bystander HepG2 cells and T98G cells significantly increased when they were co-cultured with irradiated cells or treated with the conditioned medium harvested from irradiated cells under either normoxic or hypoxic conditions. It was found that, for both cell lines, the treatment of conditioned medium and co-culture generated a similar bystander response leading to micronucleus induction. Moreover, the yields of bystander micronucleus were also similar under normoxic and hypoxic conditions. Although the hypoxic cells are radioresistant, the irradiated tumor cells can induce a similar bystander effect of micronucleus induction under different oxygen conditions. Further calculation showed that, for both cell lines, the ratio of the yield of micronucleus in bystander cells to the yield of irradiation-induced micronucleus in hypoxia was higher than that detected under normoxia (see Figures 2C,D). This result indicates that RIBE may play a more significant role in the overall radiation damage response of hypoxic cells.

### RIBE Was Induced by Free Radical Under Hypoxic Conditions

**FIGURE 3:**
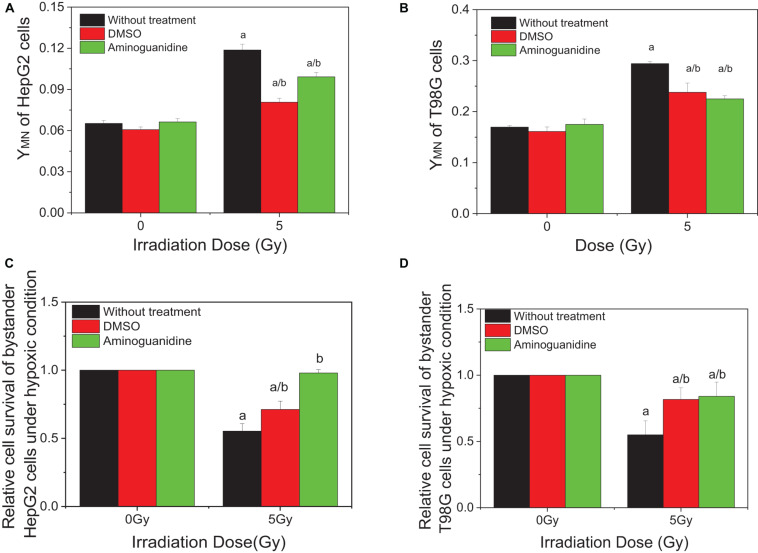
Influence of DMSO and aminoguanidine (AG) on the bystander effect in HepG2 cells and T98G cells under hypoxic conditions. Bystander tumor cells were treated with the conditioned medium harvested from irradiated conspecific cells. **(A,C)** Micronucleus induction in the bystander cells. **(B,D)** Survival fractions of bystander cells. a, *p* < 0.05 compared with the 0-Gy group; b, *p* < 0.05 compared with the 5-Gy group without chemical treatment.

Our previous studies have showed that ROS and NO contribute to the RIBE of normoxic tumor cells including HepG2 and T98G cells. To investigate whether these free radicals are involved in the RIBE in hypoxic cells, we incubated the hypoxic cells with either DMSO or AG 1 h before and during irradiation. Two hours post-irradiation, the conditioned medium without the drug was harvested and transferred to non-irradiated bystander cells that were further cultured for 24 h under hypoxic conditions until micronucleus or clonogenic assay. The representative results of bystander effects were illustrated in Figure 3 where the donor tumor cells, with or without free radical scavenger treatment, were irradiated with 5 Gy X-rays. It can be seen that, for both HepG2 and T98G cells, treatment of cells with DMSO or AG significantly reduced the yield of bystander micronucleus (Figures 3A,B) and increased the relative cell survival (Figures 3C,D) of hypoxic cells, but the yields of remaining micronucleus, or the relative cell survival, did not completely return to control levels. This may be because free radicals and other related bystander signals were scavenged or inhibited by DMSO and AG only during irradiation, as the cell culture medium was replaced, after irradiation, with a fresh medium without scavenger. Subsequent bystander signaling factors could be released from irradiated cells in the conditioned medium further inducing bystander responses.

### HIF-1α Status of Irradiated Cells Determined RIBE Under Hypoxia Conditions

**FIGURE 4:**
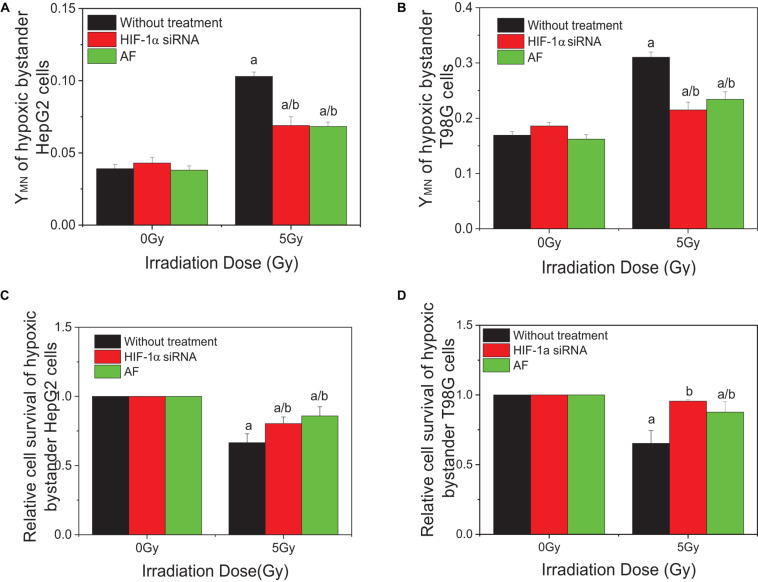
Influence of HIF-1α on the bystander effect of HepG2 cells and T98G cells under hypoxic conditions. Bystander tumor cells were treated with the conditioned medium harvested from irradiated conspecific cells, which were pretreated with HIF-1α siRNA and 5 μM 7-aminoflavone under hypoxic conditions. **(A,C)** Micronucleus induction in the bystander cells. **(B,D)** Survival fractions of bystander cells. a, *p* < 0.05 compared with the 0-Gy group; b, *p* < 0.05 compared between the 5-Gy group under different oxygen conditions.

To investigate the effect of HIF-1α on the RIBE under hypoxic conditions, cells were pretreated to inhibit HIF-1α (HIF-1α siRNA or chemical inhibitor AF) before irradiation, then harvested and the removed conditioned medium added to hypoxic bystander cells. Results showed that, for both HepG2 and T98G cells, pretreatment of irradiated cells with AF or siRNA inhibited the expression of HIF-1α and significantly reduced the yield of bystander micronucleus of hypoxic bystander cells although the yields of the remaining micronucleus were still higher than the control values (Figures 4A,B). The yields of micronucleus were decreased from 0.103 to 0.069 and 0.068 on HepG2 cells or from 0.31 to 0.215 and 0.234 on T98G cells by HIF-1α siRNA or AF treatment, respectively. Simultaneously, the relative cell survival of both hypoxic bystander cells were increased (Figures 4C,D) compared with 5 Gy without treatment; the survival fraction was increased from 0.66 to 0.804 and 0.859 in HepG2 cells or from 0.692 to 0.956 and 0.875 in T98G cells by the treatment of HIF-1α siRNA or AF, respectively. These data showed that, in hypoxic-irradiated cells, in the absence of HIF-1α expression the bystander damage in the hypoxic bystander cells was significantly reduced. It can be seen that HIF-1α also plays a key role in the RIBE under hypoxic conditions.

**FIGURE 5:**
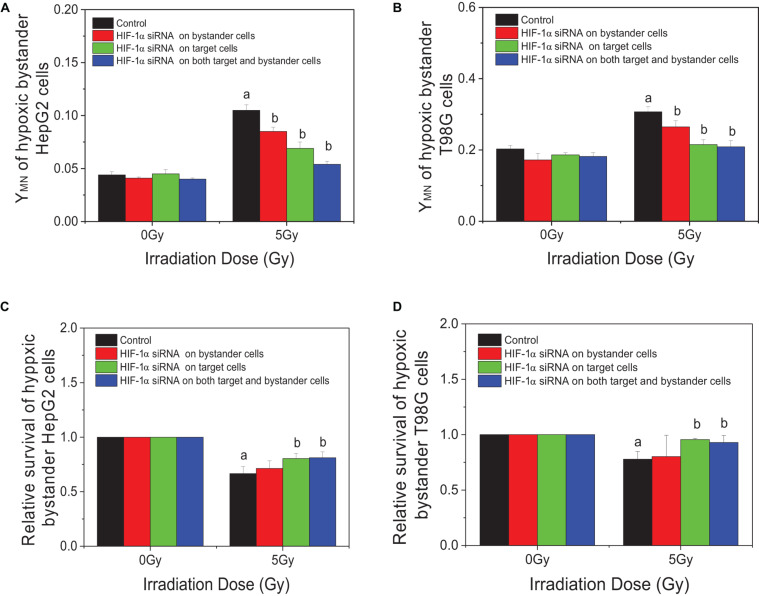
Inhibition responses of HIF-1α expression on the target (irradiated) and/or bystander HepG2 cells and T98G cells under hypoxic conditions. Four groups in this experiment: Control group, bystander cells only were treated with the conditioned-medium from irradiated conspecific cells; Inhibition of bystander cells, only bystander cells were pretreated with HIF-1α siRNA, then incubated with the conditioned medium from irradiated conspecific cells. Inhibition of target cells, only target cells pretreated with HIF-1α siRNA, then harvested and the conditioned medium transferred to bystander cells. Inhibition on both target and bystander cells, target and bystander cells were pretreated with HIF-1α siRNA, then the conditioned medium added to bystander cells. **(A,C)** Micronucleus induction in the bystander cells. **(B,D)** Survival fractions of bystander cells. a, *p* < 0.05 compared with the 0-Gy group; b, *p* < 0.05 compared with the 5-Gy group without treatment.

In order to explore the action of HIF-1α in the RIBE, both irradiated and bystander cells were pretreated with HIF1-α siRNA. Compared with irradiated controls, the yields of micronucleus of bystander cells were decreased from 0.105 to 0.085, 0.069, or 0.054 in HepG2 cells and from 0.307 to 0.265, 0.215, or 0.209 in T98G cells in three groups (bystander-inhibition group, target-inhibition group, and both bystander and target-inhibition group), respectively (Figures 5A,B). Significantly, the decrease in levels in the target-inhibition group or both target and bystander-inhibition group was higher than that of the bystander only-inhibition group. Similar results were observed for cell survival in both cell lines (shown in Figures 5C,D). The relative survival fraction was increased significantly from 0.666 to 0.804 or 0.811 for HepG2 cells and from 0.779 to 0.956 or 0.929 for T98G cells in the target-inhibition group or both target and bystander-inhibition group, respectively. Regardless of whether bystander cells were pretreated with HIF-1α siRNA, as long as target cells were pretreated with siRNA, the impact was higher than the other two groups (bystander-inhibition group and control group). Interestingly, if only bystander cells were pretreated with HIF-1α siRNA, the relative survival fraction was not markedly changed. These results confirm that HIF-1α is a key player in the RIBE detected under hypoxic conditions. It is significant that the hypoxic RIBE is regulated mainly by HIF-1α expression derived from irradiated cells, with no tight relationship with autochthonous HIF-1α expression level in hypoxic bystander cells.

### HIF-1α Was Involved in Radiation Damage in Hypoxia-Exposed Cells by Controlling Downstream Genes

**FIGURE 6:**
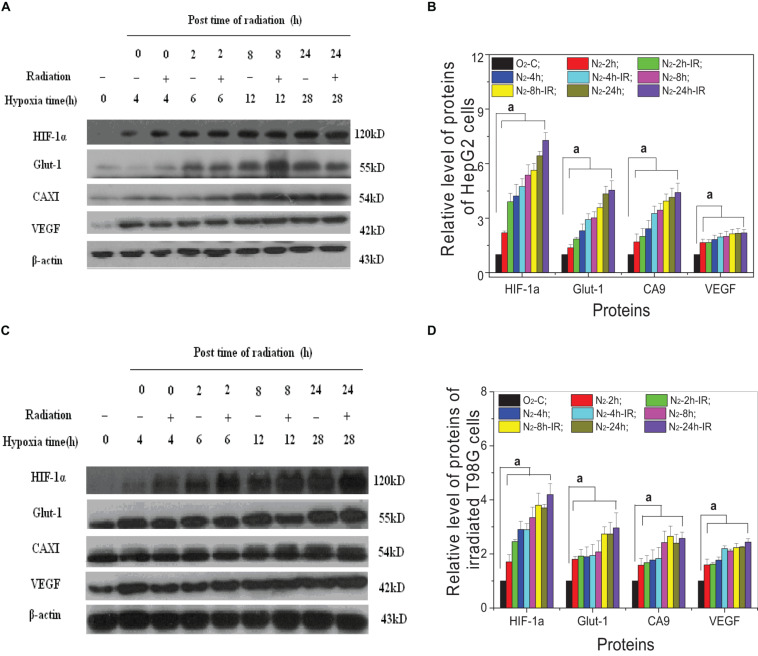
Expression of HIF-1α and its downstream proteins in irradiated hepatoma cells. Proteins were obtained from the two cell lines of HepG2 cells **(A,B)** and T98G cells **(C,D)** at 0, 2, 8, and 24 h post 5 Gy X-ray irradiation. The expression level of HIF-1α and its downstream proteins was compared with the corresponding β-actin and then normalized to that protein from cells under normoxia conditions. a, *p* < 0.05, compared with the normoxic control in the absence of irradiation.

**FIGURE 7:**
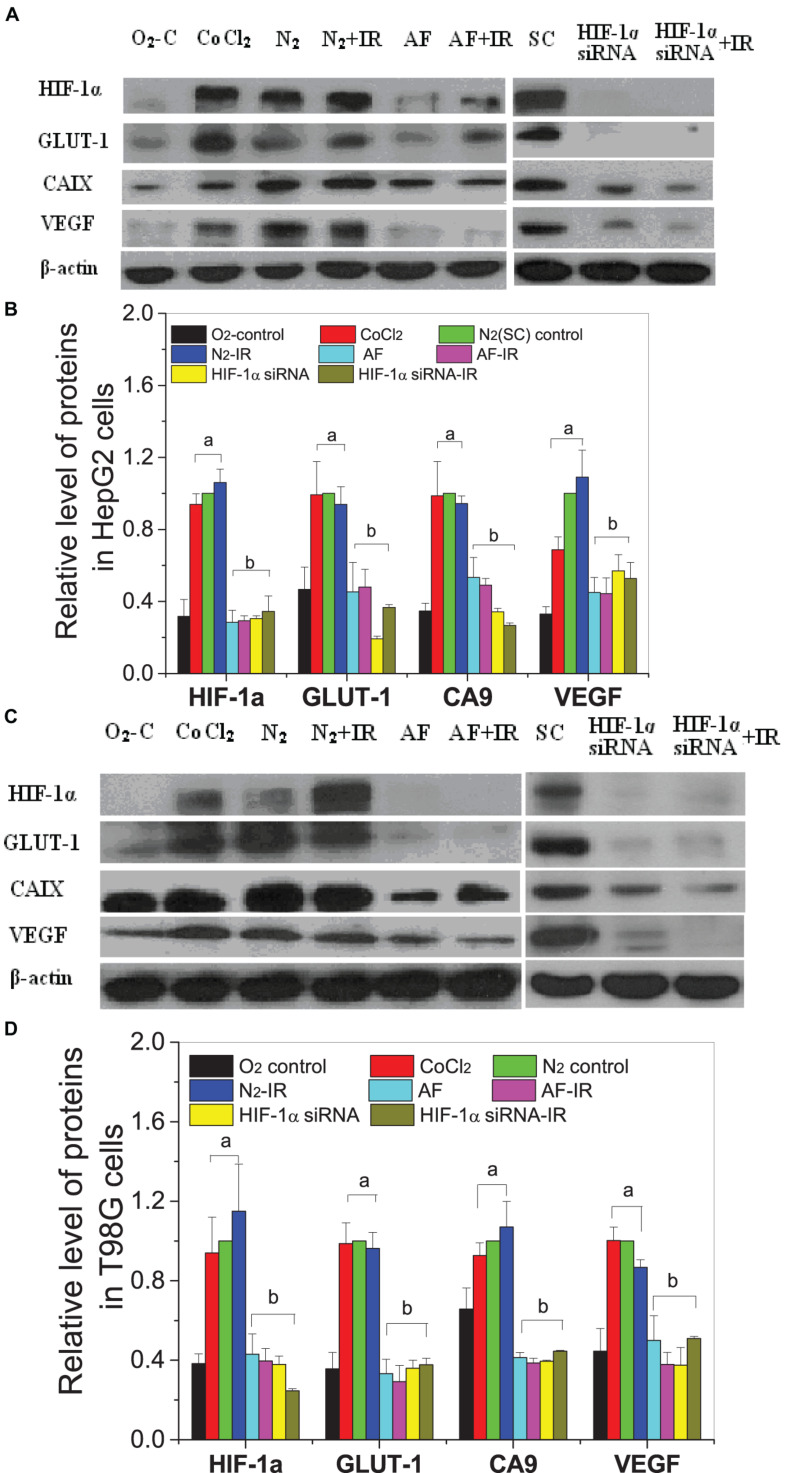
Expressions of HIF-1α and downstream proteins in hypoxic-irradiated cells pretreated with HIF-1α inhibitor treatment. The HepG2 cells **(A,C)** and T98G cells **(B,D)** were pretreated with or without 100 μM CoCl_2_, 5 μM 7-aminoflavone for 4 h, and HIF-1α siRNA for 24 h before irradiation under normoxic or hypoxic conditions, then nuclear or whole proteins were collected and analyzed at 4 h post 5 Gy X-irradiation. The HIF-1α signal and its downstream proteins were compared with the corresponding β-actin and then normalized to that protein under hypoxia. a, *p* < 0.05 compared with the normoxic control; b, *p* < 0.05 compared with the hypoxic control without irradiation.

For both HepG2 cells and T98G cells, HIF-1α expression was increased markedly in hypoxic-irradiated cells and it was also increased along with the hypoxic incubation time and post-irradiation time (Figure 6). For HepG2 cells, the expression of GLUT-1 and CA9 showed a similar trend to HIF-1α expression. However, VEGF expression was only induced by hypoxia and not by irradiation. For T98G cells, the expressions of GLUT-1, CA9, and VEGF were all increased by hypoxia treatment and were increased slightly at 8 h and 4 h after irradiation. Overall, the changes in levels of GLUT-1, CA9, and VEGF all followed their upstream gene HIF-1α. In addition, for both HepG2 cells and T98G cells, CoCl_2_ can induce an increased expression of HIF-1α to a similar extent to that produced by hypoxia (Figure 7). HIF-1α expression was markedly inhibited by both AF and HIF-1α siRNA pretreatment. Sequential irradiation did not promote HIF-1α expression under HIF-1α inhibitory conditions. The downstream expression of proteins such as CA9, GLUT-1, and VEGF was increased following the high expression of HIF-1α, either by hypoxia or with CoCl_2_, and decreased with the low expression of HIF-1α by either siRNA or AF treatment. This confirmed that HIF-1α plays an important role in the irradiation-mediated damage response under hypoxic conditions and in controlling its downstream proteins.

### Complex Mechanisms of HIF-1α Protein in Hypoxia Bystander Cells

**FIGURE 8:**
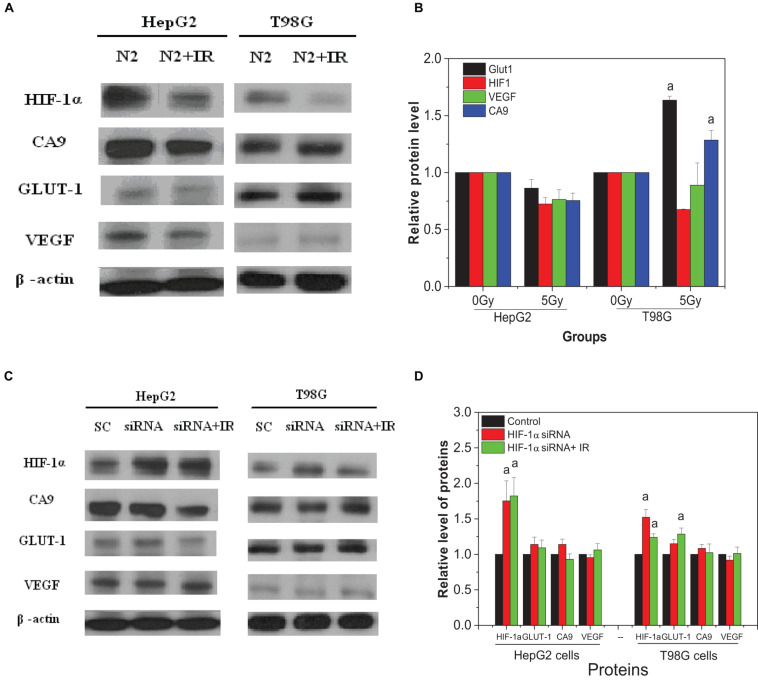
Expression of HIF-1α and related downstream proteins on the bystander cells induced by the conditioned medium under hypoxic conditions. **(A,B)** Hypoxic bystander cells were treated with the conditioned medium from hypoxic-irradiated HepG2 and T98G cells. **(C,D)** Hypoxic bystander cells were exposed to the conditioned medium from irradiated cells with or without inhibitor pretreatment. Each HIF-1α signal and its downstream protein were compared with the corresponding β-actin signal and then normalized to that protein under hypoxia conditions. a, *p* < 0.05 compared with the hypoxic control without irradiation.

Two hours after irradiation, the conditioned medium was harvested and transferred to bystander cells; 24 h later, the bystander expression of HIF-1α and downstream proteins was determined (Figures 8A,B). Interestingly, the expression of HIF-1α in hypoxic bystander HepG2 and T98G cells was significantly decreased by conditioned-medium treatment in contrast to the increase observed in directly irradiated cells. In bystander HepG2 cells, the conditioned medium also inhibited the expression of downstream signal factors VEGF and CA9, but there was no marked inhibition of GLUT-1. In contrast, to bystander T98G cells, following depressed expression of HIF-1α, VEGF was decreased slightly (*p* > 0.05), whereas CA9 and GLUT-1 were increased significantly (*p* < 0.05). In addition, the expression of bystander HIF-1α in hypoxic HepG2 cells and T98G cells was increased significantly by the conditioned medium from specific target cells which were pretreated with HIF-1α siRNA, but this had no notable impact on CA9, GLUT-1, and VEGF (Figures 8C,D). This illustrated that different cell lines have different signaling pathway responses to the conditioned medium under hypoxic conditions. In addition, the expressions of HIF-1α and downstream genes in bystander cells were mainly regulated by those signaling factors from target cells but not by the autologous hypoxic environment. It also highlights that HIF-1α plays a different role for the direct radiation damage effect and the radiation inducible bystander effect and leads to complex signal transmission in the RIBE under hypoxic conditions.

## Discussion

Hypoxia is a major barrier for effective tumor therapy as it increases a tumor’s resistance to chemotherapy and radiotherapy (Span and Bussink, 2015; Busk et al., 2020). In addition, radiation-induced bystander effects may enhance the curative efficacy of external beam radiotherapy but may also cause fatal genetic damage to non-irradiated normal cells or induce adaptive responses to non-irradiated tumor cells, adversely affecting the overall outcome of radiotherapy (Prise and O’Sullivan, 2009; Xie et al., 2016; Griffin et al., 2020a,b). This study showed that the radiosensitivity of hepatoma cells and glioblastoma cells under normoxic conditions was higher than that under hypoxic conditions (shown in Figure 1), i.e., there is a hypoxia-mediated radioresistance effect on tumor cells. Our data also demonstrate that under hypoxic conditions irradiated tumor cells can release active signaling factors into the cell culture medium leading to damage in non-irradiated bystander cells (shown in Figures 2A,B). Interestingly, the yields of micronucleus in bystander cells under different oxygen conditions are not significant different so that the ratio of bystander micronucleus to radiation directly induced micronucleus in hypoxic cells is higher than that of normoxic cells (shown in Figures 2C,D). This result may have important implications for targeted tumor radiotherapy. Since the radiosensitivity of hypoxic tumor cells is lower than that of normoxic cells, the cell killing effect of bystander response could play a key role in radiation-induced lethal effect in tumor cells. In our study, two methods, i.e., conditioned medium transfer and cell co-culture, were used for RIBE investigation and it was found that they could lead to the measurement of similar levels of bystander damage (shown in Figures 2A,B). During cell co-culture, signaling factors released from irradiated cells can diffuse freely interacting with non-irradiated bystander cells leading to the induction of bystander damage. During a few hours of cell co-culture, bystander damage can accumulate, while some damaged cells may disappear by necrosis. For the medium transfer experiment, the signaling factors released from irradiated cells are concentrated in the conditioned medium and affect the bystander cells at a defined time. Since conditioned-medium and cell-co-culture treatment leads to similar bystander responses, this suggests that the bystander signaling factors are relatively stable in the medium. Recent studies have also investigated the potential impact of modulated beams on the response of hypoxic cells to mimic the steep gradients observed with advanced radiotherapies. By using shielding strategies, bystander signaling between irradiated and non-irradiated areas of a cell culture can be compared (Butterworth et al., 2011). Similarly to what is observed here, although an oxygen dependency of directly irradiated cells was observed, under hypoxia no significant difference in the out-of-field (bystander) response was observed (Thompson et al., 2017).

It has been known that the generation of ROS and NO is an early event of RIBE on both normal cells and tumor cells under normoxic conditions (Shao et al., 2008c; He et al., 2012; Zhang et al., 2012), which is consistent with present results in Figure 3 where DMSO and AG partly suppressed the RIBE in HepG2 and T98G cells. Importantly, it is reported here that ROS and NO also contribute to the RIBE in these tumor cells under hypoxic conditions. Others also reported that ROS could be induced in the irradiated hypoxic cells. Lee et al. (2008) found that intracellular ROS was significantly increased in proton-irradiated HCT116 cells under hypoxia conditions and this contributed to radiation-induced apoptosis that could be abolished by treatment with the antioxidant N-acetyl cysteine (NAC). NO can act as an effective hypoxic cell radiosensitizer, by mimicking the effects of oxygen via fixation of radiation-induced DNA damage; however, the required levels cannot be observed *in vivo* because of vasoactive complications (De Ridder et al., 2008a,b). However, ROS and NO free radicals have very short half-lives and cannot be maintained stably in the conditioned medium, and thus they may partly play roles as bystander signaling factors by regulating other downstream cytokines (Meng et al., 2018). For instance, radiation-induced cytochrome C (Cyt-C) has a significant role in regulating bystander effect through an iNOS-triggered NO signal (He et al., 2012), and transforming growth factor β1 (TGF-β1) can be activated by NO stress in a dose- or time-dependent fashion and it can further induce bystander responses including secondary free radicals and chromosome damage (Shao et al., 2008b,c). Our data support the concept that there must be other biological mechanisms mediating a hypoxic radiation-inducible bystander effect, in addition to the free radical effect induced directly by irradiation.

Among various intrinsic and extrinsic factors, HIF-1α is the key mediator of hypoxic signal transduction, controlling the expression of more than 100 genes and exerting a diverse and complex impact on tumor radiotherapy. In response to hypoxia, HIF-1α induces VEGF and PDGF, which leads to angiogenesis and also promotes cell survival by increasing glucose transport, converting cellular metabolism to the glycolytic system (Clara et al., 2014; Bajbouj et al., 2017). HIF-1 can also induce cell-cycle arrest (Clara et al., 2014) and inhibit apoptosis signals (Sun et al., 2015), which could result in decreased radiosensitivity. Our study has shown that HIF-1α can be activated and induced not only by hypoxia but also by irradiation (Figure 6) in both HepG2 cells and T98G cells, and its expression increased following time under hypoxic and post-irradiation time. Downstream protein (VEGF, GLUT-1, and CA9) expression also increased following increased HIF-1α expression. Cells were pretreated with HIF-1α siRNA or a chemical inhibitor of HIF-1α (7-aminoflavone, AF) before irradiation, showing a significantly decreased expression of HIF-1α and its downstream proteins (shown in Figure 7). Radiosensitivity, measured using a cell survival assay (shown in Figure 1), under hypoxic conditions, was increased by suppression of HIF-1α expression. These results confirm that HIF-1α can play an important role in hypoxia-induced radioresistance. HIF-1α becomes activated in response to radiation exposure in hypoxic solid tumors and functions by protecting tumor blood vessels from the cytotoxic effects of radiation via inducing the expressions of VEGF, GLUT-1, or CA9, assuring the delivery of oxygen and nutrients to cells, and eventually accelerating tumor (Horsman et al., 2012; Towner et al., 2013). So, a blockade of activity of HIF-1 enhances the therapeutic effect of radiation.

In addition, based on our results that bystander effects play an important role in radiation-induced lethal effects of tumor cells under hypoxic conditions, greater than that under normoxic conditions, we determined the effect of HIF-1α on RIBE. When hypoxic-irradiated tumor cells were pretreated with an HIF-1α inhibitor, the DNA damage in the non-irradiated bystander cells was attenuated, while cell survival of bystander cells was increased. Interestingly, this was only observed when either irradiated only or irradiated and bystander cells were inhibited. If Hif-1α is inhibited only in hypoxic bystander cells, cell survival does not change and a decreased level of micronucleus yield is observed in comparison to inhibition of irradiated or irradiated plus bystander cells. In contrast to irradiated cells, the expression of HIF-1α in hypoxic bystander cells was decreased following the treatment with the conditioned medium from hypoxic-irradiated cells (shown in Figures 8A,B), and no changes in downstream proteins were observed. In the HepG2 cell line, the expression of CA9 and VEGF reduced coinciding with the decrease of HIF-1α, but no change was observed in GLUT-1. However, in T98G cells, there was no change in VEGF, whereas GLUT1 and CA9 were both increased significantly. When irradiated cells were pretreated with an inhibitor of HIF-1α, the expression of bystander HIF-1α was increased, but there was no change in the other three proteins (shown in Figures 8C,D). It is thus evident that the mechanism underlying a bystander effect is dependent on cell-type specificity. Overall, combining our results, it could be postulated that the contribution of HIF-1α in irradiated (directly targeted) cells is greater than that of non-irradiated bystander cells to produce a hypoxic-dependent RIBE. Also, HIF-1α plays a direct stimulating role in the hypoxia-induced radioresistance of targeted cells via regulating downstream genes but plays an adverse effect regarding a bystander effect. This result is supported by previous reports that the gene expression profiles of irradiated cells and bystander cells are significantly different and that more than 50% of the genes upregulated in irradiated cells could be downregulated in bystander cells. For example, the NF-κB, p21^*Waf1*^ (Iwakawa et al., 2008), and CSE/CBS (enzymes which endogenously synthesize H_2_S) genes were activated in irradiated cells, but not in bystander cells (Zhang et al., 2011, 2012). Downregulation of HIF-1α in bystander cells could reduce the protective effect of HIF-1α, making bystander cells more susceptible to bystander stress signaling, but not via downstream genes such as VEGF, CA9, and GLUT-1 which were quantified in our experiments.

In summary, hypoxia occurs in the microenvironment of solid tumors as a result of the complex interaction of numerous factors, including blood vessels, interstitial tissues, and tumor cells. A radiation-inducible bystander effect plays a more significant role in the overall damage response in tumor cells exposed under hypoxic conditions. As well as free radicals, such as NO and ROS, induced by irradiation, HIF-1α and its downstream-regulated proteins were involved. Under hypoxic conditions, irradiated hepatoma carcinoma HepG2 or glioblastoma T98G cells can promote the expression of HIF-1α, which would result in radioresistance, but the conditioned medium from these cells can inhibit HIF-1α expression and induce cell damage in bystander cells. These findings suggest that inhibition of HIF-1α may enhance radiosensitivity and reduce bystander cell damage, and these could help deliver potential benefits to strategies for therapeutic treatment of hypoxic tumors.

## Data Availability Statement

The raw data supporting the conclusions of this article will be made available by the authors, without undue reservation.

## Author Contributions

CS, KP, and JZ contributed to the overall conception and design of the study. GP and KB performed the acquisition and analysis of the data. YZ and FM performed further processing of the images. JZ constructed the first draft of the manuscript. CS and KP revised the manuscript prior to submission. All authors contributed to the article and approved the submitted version.

## Conflict of Interest

The authors declare that the research was conducted in the absence of any commercial or financial relationships that could be construed as a potential conflict of interest.
